# Genetic Influences on Receptive Joint Attention in Chimpanzees (*Pan troglodytes*)

**DOI:** 10.1038/srep03774

**Published:** 2014-01-20

**Authors:** William D. Hopkins, Alaine C. Keebaugh, Lisa A. Reamer, Jennifer Schaeffer, Steven J. Schapiro, Larry J. Young

**Affiliations:** 1 Neuroscience Institute and Language Research Center, Georgia State University, Atlanta, Georgia 30302; 2 Division of Developmental and Cognitive Neuroscience, Yerkes National Primate Research Center, Atlanta, Georgia 30329; 3 Center for Translational Social Neuroscience, Division of Behavioral Neuroscience and Psychiatric Disorders, Yerkes National Primate Research Center, Department of Psychiatry and Behavioral Sciences, Emory University, Atlanta, GA 30329; 4 Department of Veterinary Sciences, The University of Texas M. D. Anderson Cancer Center, Bastrop, Texas 78602

## Abstract

Despite their genetic similarity to humans, our understanding of the role of genes on cognitive traits in chimpanzees remains virtually unexplored. Here, we examined the relationship between genetic variation in the arginine vasopressin V1a receptor gene (*AVPR1A*) and social cognition in chimpanzees. Studies have shown that chimpanzees are polymorphic for a deletion in a sequence in the 5′ flanking region of the *AVPR1A*, DupB, which contains the variable RS3 repetitive element, which has been associated with variation in social behavior in humans. Results revealed that performance on the social cognition task was significantly heritable. Furthermore, males with one DupB^+^ allele performed significantly better and were more responsive to socio-communicative cues than males homozygous for the DupB- deletion. Performance on a non-social cognition task was not associated with the *AVPR1A* genotype. The collective findings show that *AVPR1A* polymorphisms are associated with individual differences in performance on a receptive joint attention task in chimpanzees.

At approximately 6 months of age, typically developing human infants begin to respond to socio-communicative cues of conspecifics such as gaze and following pointing cues. Some have suggested that the development of responding to joint attention cues is important for the emergence of language^1^ and other socio-cognitive abilities later in life such as social learning^2^, theory-of-mind and attribution of mental states^3^,^4^,^5^,^6^. For example, Brooks and Meltzoff^7^ examined gaze following and responses to adult pointing in 10-month-old infants and found that performance predicted vocabulary at 2 years of age see also^8^,^9^,^10^. Deficits in responding to joint attention cues early in life have also been linked to some pervasive developmental disorders, notably autism spectrum disorder (ASD)^11^,^12^,^13^ as well as specific language impairments^14^.

Not only do children respond to gaze and manual pointing, but studies in nonhuman primates, including monkeys but particularly great apes, have shown some similar findings. A number of nonhuman primate species will follow the gaze of human experimenters or conspecifics^15^,^16^,^17^. There is also evidence that chimpanzees and other great apes will orient to manual gesture cues and, depending on their rearing experiences, can select baited objects on the basis of human pointing cues^18^,^19^,^20^. Thus, comparatively, responding to gaze and manual pointing cues seem to be a shared trait among primates and may have a strong evolutionary foundation.

In studies of both human and nonhuman primates, there are considerable individual differences in receptive joint attention (RJA) performance; however, the potential biological factors that underlie this variation are poorly understood^10^,^21^. Though some studies in apes suggest that different rearing experiences can influence performance on RJA tasks^19^,^20^, the potential role that genetic factors might play remains largely unknown and unexplored. Liu et al. (2001) identified one genetic locus (11g23) that was related to measures of joint attention taken from the Autism Diagnostic Interview –Revised test in a large sample of individuals with a diagnosis of ASD. Two studies have also shown that some of the traits associated with autism such as socio-communicative competence, as measured by parent questionnaires, are significantly heritable^22^,^23^. Despite the long standing evidence of genetic similarities between humans and chimpanzees, there are very few studies that have examined the role of genes on social behavior and cognition in apes^24^,^25^. Here, we sought to examine the potential role that genetic factors might play in determining individual variation in RJA performance in chimpanzees.

Specifically, we assessed RJA performance in a sample of captive chimpanzees for which there is a well-documented pedigree. In the initial analysis, we capitalized on the existence of the pedigrees to perform a quantitative genetic analysis in order to estimate heritability in RJA performance of the chimpanzees. Similar methods have recently been employed in several nonhuman primate species to determine potential genetic contributions to individual differences in behavioral traits such as a novelty-seeking^26^, arousal and anxiety^27^,^28^, and sociality^29^. We hypothesized that if individual differences in RJA performance have a potential genetic basis, then significant heritability in performance would be found within the chimpanzee sample.

In addition to the quantitative genetic analysis, we also assessed the potential influence of a vasopressin receptor gene (*AVPR1A*) on RJA performance in the chimpanzees. The *AVPR1A* gene was selected as a candidate gene for several reasons. Notably, vasopressin is a neuropeptide with multiple physiological functions that has been strongly implicated in the development and evolution of complex social relations and cognition in mammals^30^,^31^. Studies in several species have shown that one of three known AVP receptors, arginine vasopressin V1a receptor (AVPR1A), is expressed in the brain and plays a prominent role in producing a range of social behaviors. For example, meadow and prairie voles, which differ dramatically in their pair bonding behavior, show pronounced differences in AVPR1A distribution in the brain^32^. More recently, several studies in voles have examined variation in behavior and AVPR1A expression in relation to a repetitive polymorphism referred to as a microsatellite in the 5′ flanking region of the AVPR1A gene (*Avpr1a*). There are both individual and species differences in *Avpr1a* microsatellite structure, which has been associated with variation in AVPR1A expression in the brain, pair bonding and other dimensions of social behavior in voles, particularly males^32^,^33^,^34^,^35^,^36^. Furthermore, individual variation in vole Avpr1a microsatellite structure has been demonstrated to causally mediate variation in AVPR1A binding in the brains of knock-in mice^37^.

These studies parallel findings in humans that describe an association between microsatellite variation in the *AVPR1A* and social behavior^38^. For example, the *AVPR1A* microsatellite polymorphism, RS3, is a complex (CT)_4_-TT-(CT)_8_-(GT)_24_ repeat that is 3625 bp upstream of the transcription start site. Homozygosity in allele 334 of RS3 in men (but not women) is associated with variation in pair bonding behavior, as measured by traits such as relationship quality, perceived marital problems, marital status, and spousal perception of marital quality^39^. In a study of university students, participants with short (308–325 bp) versus long (327–342) versions of RS3 were less generous, as measured by the dictator game and by self-report. Although the molecular mechanisms underlying this association are not known, long alleles of RS3 are associated with increased *AVPR1A* mRNA in postmortem hippocampal tissue compared to shorter alleles^40^. As the RS3 lies in the 5′ flanking region of the gene, it is likely that variation in RS3, or a linked functional polymorphism, results in variation in *AVPR1A* expression in specific brain regions, as previously demonstrated in rodents^37^. Indeed, variation in RS3 sequence has been shown to influence promoter activity in a cell culture-based transcription reporter assay^41^. In sum, given the importance of this gene in the social behavior of humans and nonhumans, we reasoned that it might be involved in basic socio-communicative skills that facilitate the formation of social relationships and socio-communication skills, such as RJA.

Additionally, chimpanzees are an excellent model to explore the functional role of the RS3 polymorphism in the *AVPR1A* gene because there is a common indel (insertion/deletion) resulting in a complete deletion of the RS3 sequence in roughly 65% of chimpanzees^42^,^43^. In humans, the RS3 repeat region is housed within a larger, ~350 bp tandem duplicated region. The first of these duplicated regions, DupA, spans −3730 to −4074 bp relative to the transcription start site and contains a GT_20–26_ microsatellite, known as STR1. The second block, DupB, spans −3382 to −3729 bp and contains the complex microsatellite, RS3 ((CT)_6–14_(GT)_8–24_). Chimpanzees are polymorphic for the presence of the RS3-containing DupB region (DupB^+^), leading to a 357 bp difference between the DupB^+^ and DupB^−^ alleles^42^. The deletion of RS3 in **~**65% of chimpanzees makes this species ideal for assessing the potential role of the *AVPR1A* gene, and more specifically RS3, on social behavior and cognition. Finally, it has been previously reported that DupB^+/−^ male chimpanzees are rated as more dominant than DupB^−/−^ males^25^. One interpretation of this finding was that DupB^+/−^ males might be more dominant because they are more sensitive to subtle, non-verbal social cues produced by conspecifics that would presumably result in them responding more appropriately than those who are less sensitive (DupB^−/−^). This, in turn, might facilitate the formation of reciprocal relationships among group members, thereby increasing the social support needed to raise social status within the group as well as potentially attract more mates^44^,^45^. In short, increased sensitivity to socio-communicative cues might provide some adaptive advantages in terms of potentially attracting mates and avoiding conflict with conspecifics.

To this end, we genotyped a sample of captive chimpanzees for the *AVPR1A* DupB region containing RS3 and also tested them on a RJA task, originally developed for use with typically developing human children and those at risk for developmental disorders, such as autism^46^. We hypothesized that if variation in RS3 and *AVPR1A* expression contributes to individual differences in RJA performance, then heterozygous DupB^+/−^ chimpanzees would perform better than homozygous DupB^−/−^ genotype individuals.

## Results

### General RJA performance

In the initial analysis, we examined whether the chimpanzees were more likely to respond in the experimental compared to baseline conditions as a means of assessing the effectiveness of this test to elicit RJA responses and to rule out the possibility that the apes were randomly responding. For this analysis, we performed a Wilcoxon signed rank test based on the summed scores for the two baseline and two test trials and a significant difference was found between conditions (*z* = 12.22, N = 232, *p* < .001). Eighty-two percent of the chimpanzees (N = 190) performed better on the experimental compared to baseline test whereas the remaining18% of the chimpanzees (N = 42) responded equally or did worse on the experimental compared to the baseline tests. The distribution of performance scores for males and females is presented in Table 1.

### Quantitative genetic analysis

**Figure 1:**
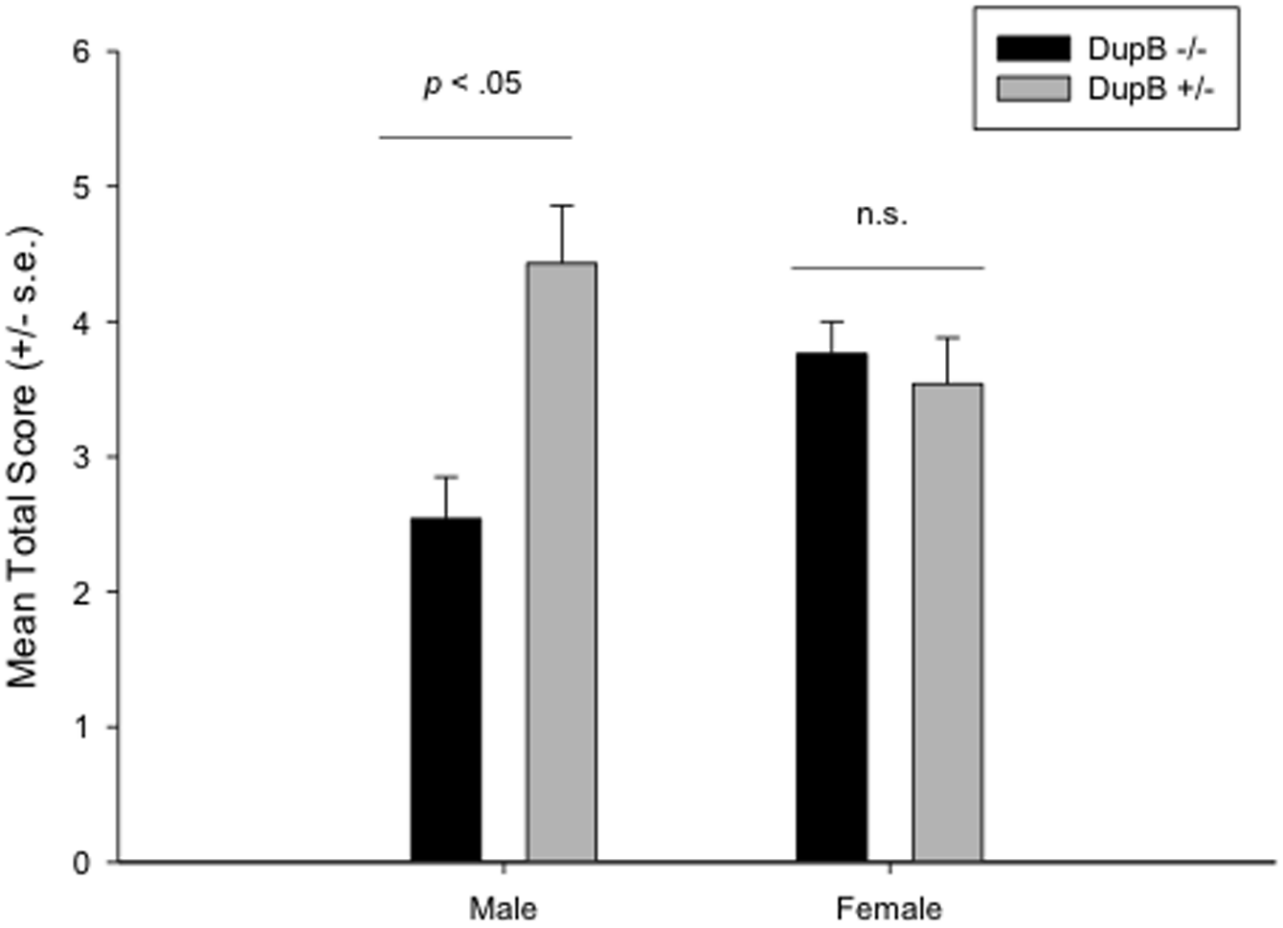
Mean RJA Score (+/− s.e.) for DupB +/− and DupB −/− Male and Female Chimpanzees.

We next performed the initial estimation of h^2^ in RJA performance for the entire sample. RJA performance was found to be significantly heritable in the chimpanzees (h^2^ = .252, s.e. = .126, *p* < .01). We next ran the quantitative genetic analysis again but added age, colony (YNPRC, UTMDACC), sex (male, female), *AVPR1A* genotype (DupB−/−, DupB−/+) and the sex X *AVPR1A* interaction terms as covariates in the model. With these variables in the model, heritability in RJA performance was reduced and only borderline significant (h^*2*^ = .228, s.e. = .152, *p* < .06). The covariates sex, *AVPR1A* and the interaction between sex and *AVPR1A* genotype were all significant and accounted for 4.9% of the variance. Because the higher order sex by *AVPR1A* interaction was significant, we focused the post-hoc interpretation of this finding. As can be seen in Figure 1, DupB^+/−^ males needed significantly fewer social cues than DupB^−/−^ males to elicit an orienting response. No significant differences were found between DupB^+/−^ and DupB^−/−^ females.

As a secondary analysis, we performed a more traditional analysis of variance with the RJA score serving as the dependent measure while colony (YNPRC, UTMDACC) sex (male, female) and *AVPR1A* genotype (DupB−/−, DupB+/−) were fixed factors. This analysis revealed a significant two-way interaction between sex and *AVPR1a* genotype *F*(1,205) = 6.217, *p* < .02. Post-hoc analysis indicated that DupB^+/−^ males needed significantly fewer social cues than DupB^−/−^ males to elicit an orienting response. No significant differences were found between DupB^+/−^ and DupB^−/−^ females. No other significant main effects or interactions were found.

### Spatial memory

Recall that this task was administered only to the 71 YNPRC chimpanzees and was used to evaluate whether the observed *AVPR1A* effects on RJA were specific to that task or were also evident for other non-social tasks. Thus, we examined the effects of *AVPR1A* genotype and sex on SM and RJA performance using MANOVA. RJA and SM performance were the dependent measures while sex (male,female) and *AVPR1A* (DupB−/−, DupB+/−) were the independent variables. The MANOVA revealed a significant two-way interaction between sex and *AVPR1A* *F*(2, 66) = 2.87, *p* < .05. Subsequent univariate F-test indicated that the significant interaction between sex and *AVPR1A* was specific to the RJA *F*(1, 54) = 4.40, *p* < .04 and not the SM task *F*(1, 66) = .530, *p* = .37. The mean RJA and SM performance in DupB−/− and DupB−/+ male and female chimpanzees are shown in Table 2. As was the case on the overall analysis of RJA performance, Dup+/− males did significantly better on the RJA task compared to DupB−/− males. No significant differences in RJA task performance were found between DupB−/− and DupB+/− females.

## Discussion

Three findings emerged from this study. First, consistent with previous studies, chimpanzees orient to human communicative cues including gaze and pointing and do not in their absence. Second, individual differences in RJA performance were significantly heritable, suggesting that genetic variation explains 25% of the variation in RJA performance. Third, after controlling for genetic relatedness, RJA performance was linked to variation in the *AVPR1A* gene and this was particularly the case for male but not female chimpanzees. Our analysis suggests that variation in *AVPR1A* RS3 explains approximately 5% of the variance in RJA performance, which is a fairly robust effect size for a single gene's impact on behavior. While our data do not suggest that variation in *AVPR1A* contributes to a large proportion of individual variation in RJA, the data do suggest that AVPR1A plays a role in the socio-communicative skills of chimpanzees. Indeed, these data are the first evidence of a genetic basis for explaining individual differences in RJA performance in any species, including humans (but see^21^). It should be emphasized that the influence of the RS3 polymorphism in the chimpanzees was specific to RJA skills and was not related to the spatial memory task, at least in the YNPRC chimpanzees (see Table 2). Furthermore, because we failed to find a main effect or significant interaction between colony and AVPR1A genotype, it shows the influence of the *AVPR1A* gene on RJA performance was evident in two, independent samples of chimpanzees (YNPRC and UTMDACC).

The overall results suggest that the *AVPR1A* gene influences the sensitivity in perception of socio-communicative cues by male but not female chimpanzees. As RS3 has been linked to *AVPR1A* mRNA expression in human post-mortem tissue, and promoter activity in transcription assays^41^, these data suggest that variation in expression in the brain, rather than protein structure, contributes to individual differences in RJA performance. The sex specific influence of the *AVPR1A* gene on social cognition has not been previously reported in human or nonhuman primates but is consistent with studies demonstrating that vasopressin has selective effects on male social behavior in voles, including pair bonding^32^. These sex differences are likely mediated in part by the sexually dimorphic expression of vasopressin (AVP), the ligand for AVPR1A in extrahypothalamic brain regions^47^. As males have higher levels of AVP than females, one would expect sex-specific effects of variation in AVPR1A expression on behavior, as previously reported in mice^48^.

In humans, the RS3 in the *AVPR1A* has been linked to variation in relationship quality in men but not women^39^. Sex specific effects of intranasal vasopressin administration on social communication have been reported in humans. In men, vasopressin stimulated agonistic facial motor patterns in response to the faces of unfamiliar men and decreased perceptions of the friendliness of those faces, while in women, vasopressin stimulated affiliative facial motor patterns in response to the faces of unfamiliar women and increased perceptions of the friendliness of those faces^49^. Furthermore, there is evidence that intranasal administration of vasopressin in males results in greater cooperative behavior in response to a communicative gesture within the Prisoner's dilemma game, a task that presumably measures reciprocal altruism^50^. Our results suggest that chimpanzees carrying the DupB^+^ allele with RS3, which is similar to the human *AVPR1A* gene structure, are more sensitive, and respond better to, socio-communicative cues than those individuals homozygous for the more common DupB^−^ allele, which is missing the RS3 microsatellite.

There are at least three limitations to this study. First, we examined the heritability and role of the *AVPR1A* on RJA performance in the chimpanzees. Though we compared both RJA and SM performance within the YNPRC cohort, data on additional non-social cognitive measures in all the apes would be more desirable. Notwithstanding, we do believe that the effect of the *AVPR1a* polymorphism reported here likely does reflect its role in subject abilities to attend to socio-communicative cues rather than more general intellectual skills for two reasons. First, as noted above, there is a fairly large body of research implicating the *AVPR1A* gene in a variety of social behaviors in mammals and the results reported are consistent with these observations. Further, some preliminary analyses examining the effect of the *AVPR1A* gene on a different non-social cognitive task in a subset of chimpanzees in this study revealed little evidence that it influences performance. Specifically, within the UTMDACC and YNPRC cohort, we have previously assessed cognition for a variety of different problem solving and motivational tasks. For instance, we provided 145 chimpanzees that were genotyped for the *AVPR1A* with coconuts and measured whether they were able to successfully learn to open them within three separate test sessions. Performance on the coconut task was found to be heritable (h^2^ = .80, *p* < .001) but *AVPR1A* genotype was not significantly associated with performance with 67% (43/59) and 66% (46/76) percent of the DupB^−/+^ and DupB^−/−^ chimpanzees able to solve the problem. Separate analyses of the association between *AVPR1A* genotype and success in opening a coconut for males and females showed no significant effects for either sex. Among the males, 74% (32/43) and 69% (11/16) percent of the DupB^−/−^ and DupB ^−/+^ individuals could solve the task. Likewise, in the females, 59% (31/53) and 65% (15/23) of the DupB ^−/−^ and DupB ^−/+^ subjects successfully opened the coconut. Thus, like the SM task, these data do not support the notion that the *AVPR1A* is generally involved in problem solving abilities.

A second, limitation was the inability to obtain a reasonable sample of chimpanzees with the DupB ^+/+^ genotype for inclusion in the genotype analysis. As noted in the methods section, we identified only 9 chimpanzees with the DupB+/+ genotype including 8 females and 1 male. The limited number of DupB+/+ chimpanzees is consistent with previous findings by Donaldsen et al.^42^ showing that this genotype represents only about 3% of the distribution and is clearly the minority genotype. Any attempt to include this cohort in our sample would have been problematic because of the small sample and highly skewed nature of the genotype between females and males. Thus, we can infer that differences are evident between the DupB^−/−^ and DupB^−/+^ individuals but how the DupB^+/+^ individual would perform on the RJA task in comparison is unknown and will require additional data collection from another sample of chimpanzees.

Third, this study showed that receptive joint attention was associated with the absence or presence of the DupB+ in our chimpanzee sample. As such, this should be considered a first step in a larger attempt to understand the role of the AVPR1A on socio-communicative and cognitive process in chimpanzees; however, we recognize that the variation in DupB+ may be in disequilibrium with another functional elements and we cannot rule this out based on the findings of this study. We also know of no data on where *AVPR1A* is expressed in the chimpanzee brain and how this might interact with neurobiological measures to explain individual differences in RJA performance. For instance, Hopkins and Taglialatela^51^,^52^ have previously found that initiating joint attention is associated with variation in grey matter volume within the anterior cingulate cortex while RJA and gaze following in chimpanzees are both associated with individual differences in grey matter volume and asymmetry in the superior temporal gyrus. Based on these findings, one might predict that *AVPR1A* may be expressed in the anterior cingulate cortex and superior temporal gyrus (among other regions), as has been reported in rhesus macaques^53^. Alternatively, studies examining gene expression using *in vitro* assays in relation to microsatellite variability in cell culture for different cell types would be potentially fruitful. These techniques have been used in humans and rodents^35^,^41^,^54^ and in at least one study on the AVPR1A in chimpanzees, though these authors assessed a different polymorphism (RS1) than the RS3 examined in this paper^63^. Clearly these issues warrant further exploration.

Lastly, as is the case in any genotype-phenotype study, our findings should be interpreted within the context of potential false positive results. By limiting our analyses to a single dichotomous polymorphism in the *AVPR1A* in relation to finite set of tasks, we have attempted to limit the potential for Type I error and thereby minimize reporting a potential false positive. Furthermore, as noted above, because we found no significant main effects or interactions for the colony variable in relation to sex or AVPR1A genotype, we believe this represents an independent replication of the results between two chimpanzee samples. Notwithstanding, additional studies are needed to determine the consistency of the results reported here in other chimpanzee populations.

In a larger evolutionary context, the findings reported here are consistent with the hypothesis that higher ranking chimpanzees may be more sensitive to interpreting and responding to social cues than lower ranking individuals. Recall that Hopkins et al.^25^ reported that DupB^+/−^ males were ranked as more dominant than DupB ^−/−^ males and they hypothesized that these individuals, in turn, may be dominant because of their sensitivity in perceiving and responding to different social cues. Based on these previous findings, combined with those reported here, it leads to the hypothesis that significant associations would be found between social rank and RJA performance in chimpanzees. It further suggests that the association between social rank or dominance and RJA performance would be mediated by variation in the DupB region in chimpanzees.

Finally, it has been hypothesized that the *AVPR1A* gene may be a risk factor for the development of autism-spectrum disorder (ASD)^55^,^56^. Poor initiation and response to joint attention social cues in developing children, such as gaze following and pointing, have been described as potential behavioral risks factors for the development of ASD^13^,^57^. Our findings linking individual difference in responding to joint attention within the DupB region in male chimpanzees suggest that the *AVPR1A* gene should be further investigated as a potential genetic marker for explaining individual differences in RJA performance and potentially socio-cognitive endophenotypes of ASD subjects, particularly males.

## Methods

### Subjects

The quantitative genetic analysis for the RJA task was based on a sample of 232 adult or sub-adult chimpanzees and all individuals were residing at either the Yerkes National Primate Research Center (YNPRC, n = 71) or The University of Texas M. D. Anderson Cancer Center (UTMDACC, n = 161). Based on previous analyses of the composition of subspecies of chimpanzees in captive United States populations, we assumed that at least 95% of our sample was represented by *Pan troglodytes verus*^58^. The chimpanzees lived in social groups between 2 and 22 individuals and received daily diet of fruits, vegetables and chow. Within the total sample of 232 chimpanzees, behavioral and genotype data were available in 213 including 132 females and 81 males. All procedures were approved by the appropriate Animal Care and Use Committees and followed the Institute of Medicine guidelines on the ethical use of chimpanzees in biomedical and behavioral research.

### Procedure (behavioral test)

#### Receptive joint attention (RJA)

**Figure 2:**
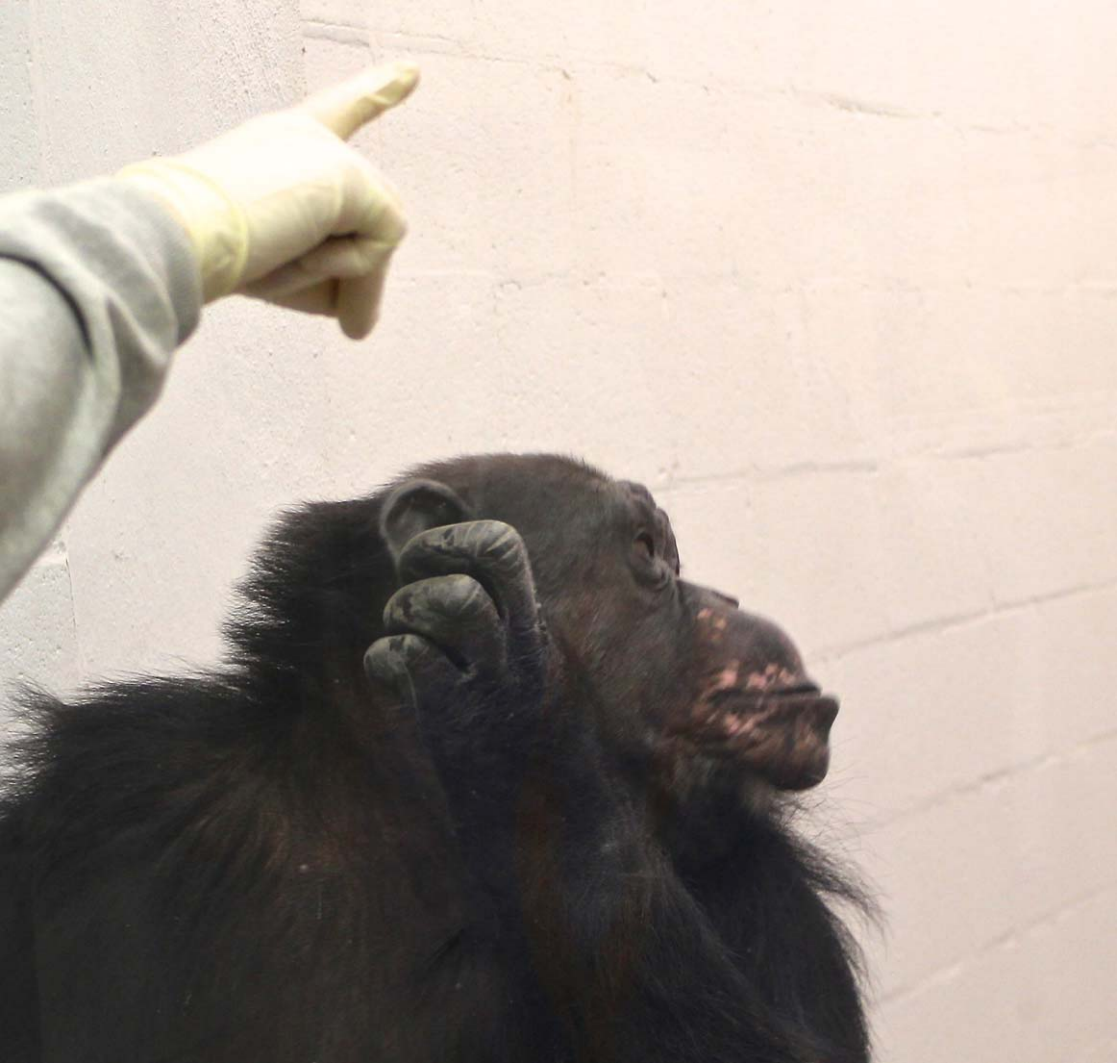
Photograph of a chimpanzee orienting in response to a gaze and pointing cue from the experimenter. Photo was taken by Jennifer Schaeffer.

The RJA task was modeled after a similar measure employed with typical and atypical developing human children by Dawson et al.^46^ that assesses both a) overall sensitivity to socio-communicative cues by human experimenters, including gaze and manual pointing and b) the number of social cues needed to elicit an orienting response from the apes. To test for RJA, at the start of a trial and between each step of the procedure, the experimenter sat in front of the subject and engaged them in a basic husbandry activity such as a body exam (i.e., simple commands such as presenting lips, leg, arm, etc.). When the subject was actively engaged with the experimenter, test trials began. For each step of the test trials, the experimenter re-engaged the subject before performing the trial. In between each step of the test, the experimenter (E) continued to engage the subject in the husbandry activity. During Step 1, E gazed for 5 sec toward a spot over and behind the subject's head. E then returned to a neutral position for 10 seconds. If the subject overtly oriented and/or looked behind them and the directed line of sight of E within the 15 second test time-frame, they receive a score of 3 for the trial and the session was ended. If the subject did not orient within the 15 second test time-frame, the subject was tested on Step 2. In Step 2, E looked at the same spot as in Step 1 but this time both gazed and pointed to the spot behind the subject for 5 seconds (as an example, see Figure 2). E then returned to a neutral position for 10 seconds. If the subject overtly oriented and/or looked in E's directed line of sight or gesture within the 15 second test time-frame they received a score of 2 for the trial and the session ended. If the subject did not orient within the 15 second test time-frame, the subject was tested on Step 3. For Step 3, E gazed and pointed at the spot behind them while saying the subjects name for 5 seconds. The experimenter could say the subjects name more than once but no more than three times within the 5 second time frame. E then returned to a neutral position for 10 seconds. If the subject overtly oriented and/or looked in the experimenters directed line of sight or gesture within the 15 second test time-frame they received a score of 1 for the trial and the session was complete. If the subject never oriented or looked within the 15 second test time-frame at the end of step 3, then they receive a score of 0 for never orienting during the test trials. Each subject was tested on the experimental task on two occasions and RJA performance was tested in the chimpanzee samples from both the YNPRC and UTMDACC.

Each subject also received two baseline trials to assess how often the apes might look behind them in the absence of any socio-communicative cues from the experimenter. During baseline test, E engaged with the subjects in the husbandry behaviors as previously described. When the apes appeared engaged, E stopped engaging and looked straight at the subject for 5 seconds, followed by the 15 second response period. If the subject's oriented or looked behind them during the response period, they were given a score of 1. If the subjects did not orient or look behind them during the response period, they received a score of 0. Prior to the start of data collection, inter-rater reliability was established between the two experimenters in the coding of orienting responses with Cohen's kappa *r* = .83, which is considered excellent. One experimenter scored the chimpanzees behavior at UTMDACC and the other experimenter scored the subjects at the YNPRC. The RJA data were collected real time and at the completion of each trial.

#### Spatial memory (SM)

This test assessed subjects' ability to remember the locations of baited food rewards and therefore was designed to assess non-social cognition^17^,^20^,^59^. During each trial, the subject was positioned in front of a testing apparatus positioned outside the subject's home cage. The experimenter sat behind the test apparatus. On each trial, the subject watched as food was hidden under small opaque containers in two of three possible spatial locations. Each subject received all three possible combinations of baited locations. The tray with the baited object was then presented to the subject and they were allowed to search the locations. The subject was scored as successful if he/she located both food items without searching in the unbaited location. Each subject received two test sessions, each comprised of three trials, for a total of 6 trials. The SM task was administered only to the seventy-one YNPRC chimpanzees.

### Data analysis

We characterized the subject's RJA performance two ways. First, we initially sought to examine overall RJA performance. For this measure, in each test trial, subjects who scored a 1, 2 or 3 were classified as passing the test. Those that received a score of 0 were classified as failing the test. Thus, subjects could pass or fail each trial with a maximum score of 2 (passed both trials) or minimum score of 0 (failed both trials). Similarly, for the baseline trials, the subjects were classified as passing or failing each trial resulting in a range of scores from 0 (never looked with no cue given) to 2 (looked both times when no cue was given). For the second performance characterization measure, we considered the raw scores on the two test trials, which could range from 6 (responded to gaze alone on both trials) to 0 (failed to respond on both experimental trials). Scores between 1 and 6 indicated some variation in the number of social cues needed to elicit an orienting response with higher scores indicating the need for fewer socio-communicative cues. For the SM task, the percentage of correct trials (out of 6) was the dependent measure.

### Quantitative genetic analysis

Total additive genetic variance (h^2^) is the proportion of total phenotypic variance that is attributable to all genetic sources. Total phenotypic variance is constrained to a value of 1; therefore, all non-genetic contributions to the phenotype are equal to 1 - h^2^. Many of the chimpanzees in each colony are related and this allowed for an analysis of heritability using quantitative genetics based on the entire pedigree. To estimate heritability in RJA performance, we used the software package SOLAR^60^. SOLAR uses a variance components approach to estimate the polygenic component of variance when considering the entire pedigree (see^61^,^62^,^63^). We used SOLAR in two ways in this study. First, we used it to estimate and statistically determine whether RJA performance was significantly heritable in the chimpanzees. Second, because we had many related individuals in our sample, we used the covariate functions within SOLAR to estimate the influence of sex and the *AVPR1A* polymorphism on RJA performance when controlling for relatedness among the individuals. Similar methods have been used to examine, for example, whether polymorphisms in the 5-HIAA serotonin transporter gene influence temperament in genetically related rhesus monkeys^28^. In this analysis, we initially estimated that heritability (h^2^) in RJA performance. When then subsequently examined changes in h^2^ when adding colony, sex, AVPR1A genotype and the interaction between these two variables as covariates in the analysis. No change or reductions in h^2^ after inclusion of the covariates indicates that the specific genes have a significant impact on the phenotype independent of the relatedness among the individuals^60^.

### DNA extraction, genotyping, and analysis

DNA samples were isolated from blood samples using Puregene DNA Purification system (Gentra, Minneapolis, MN) as described by Donaldson et al^42^. Samples were tracked via a secure Filemaker Pro 8 database that linked sample codes for each aliquot, demographics for each subject (e.g., subject number, birth date, sire, dam, etc.), DNA quantification and purity analysis results, and genotype data.

Each individual was genotyped for the *AVPR1A* DupA/B region using the primers and conditions reported in previous studies with slight modifications^42^. Briefly, we used forward primer 5′- CATACACATGGAAAGCACCTAA-3′ and a reverse primer of 5′- GCATGGTAGCCTCTCTTTAAT-3′ with an annealing temperature of 57°C for 30 cycles: 95°C, 5 min; 30 × (95°C, 30 sec; 57°C, 3 min; 72°C, 3.5 min) 72°C, 10 min; 4°C, hold. PCR amplification was undertaken using the Epicentre Failsafe kit using premix I (Illumina Inc., Madison, WI) according to the manufacturer's directions. Genotyping was performed in a volume of 25 microliters containing 20 ng target genomic DNA. PCR products were resolved on a 2% agarose gel (SeaKemAgarose LE, Lonza, Basel, Switzerland) at 120 V for 120 min with a 100 bp DNA ladder (New England Biolabs, Ipswich, MA) in TBE. The DupB containing allele resulted in a band of **~**900 bp, while the DupB minus allele was **~**570 bp long, and genotypes were visually assigned^42^. Each sample was genotyped twice with two independent PCR reactions and confirmed with separate gel analysis before the data set was finalized (N = 222). Within the entire sample, there were 147 DupB^−/−^ and 66 DupB^−/+^ and 9 DupB+/+ chimpanzees. We would note that we identified 9 DupB^+/+^ animals, however the sex ratio was severely skewed for unknown reasons (8 females and 1 male). We would further add that the distribution of DupB−/−, DupB −/+ and DupB +/+ from a sample of 47 wild caught chimpanzees genotyped by Donaldson et al.^42^ were 64%, 30% and 6%, respectively. Within our sample of captive born individuals, the distribution of DupB−/−, DupB −/+ and DupB +/+ individual was 66%, 30%, and 4%, which does not differ from those reported in the genetically unrelated wild chimpanzees X^2^(2, *N* = 269) = 0.51, *p* = .75. Thus, our genotype distribution does not deviate from Hardy-Weinberg equilibrium. Because we had so few and such a skewed sex distribution of DupB^+/+^ apes, they were omitted from the analysis presented here. However, it should be noted that when we combined DupB^+/+^ animals with DupB^−/+^, the statistical findings were not changed.

## Author Contributions

W.D.H. designed, analyzed and wrote the manuscript. A.K. and L.Y. assisted in data collection, data analysis and writing of the manuscript. L.R. and J.S. assisted in data collection and S.S. assisted in writing. All authors have read the manuscript and have agreed to the content.

## Figures and Tables

**Table 1:** Frequency Distribution in RJA Performance Scores in Male and Female Chimpanzees

	Difference Score (Test – Baseline)		
	0	1	2
Males	20	34	34
Females	22	57	65
Total	42	91	99

Note: It was possible to have a negative difference score, for example, if a chimpanzee oriented more often in the baseline compared to the test condition. This occurred in three subjects and these individuals were given a score of zero to reflect that they failed the test.

**Table 2:** Mean RJA and SM Performance in DupB+/− and DupB−/− Male and Female Chimpanzees at the YNPRC

	Males		Females	
	RJA	SM	RJA	SM
DupB−/−	2.45	57.6	4.32	63.9
s.e.	(.55)	(6.3)	(.37)	(4.3)
DupB+/−	3.67	69.4	2.88	57.8
s.e.	(.75)	(8.5)	(.45)	(5.1)

RJA = receptive joint attention, SM = spatial memory task.
